# Updating understanding of real-world adverse events associated with omeprazole

**DOI:** 10.1371/journal.pone.0330509

**Published:** 2025-08-20

**Authors:** Jijun Zhang, Jie An

**Affiliations:** 1 Third Hospital of Shanxi Medical University, Shanxi Bethune Hospital, Shanxi Academy of Medical Sciences, Tongji Shanxi Hospital, Taiyuan, China; 2 Department of General Surgery, Shanxi Bethune Hospital, Shanxi Academy of Medical Sciences, Tongji Shanxi Hospital, Third Hospital of Shanxi Medical University, Taiyuan, China; Icahn School of Medicine at Mount Sinai Department of Pharmacological Sciences, UNITED STATES OF AMERICA

## Abstract

**Background:**

This study aims to assess the adverse events (AEs) and safety profile of omeprazole, a widely used proton pump inhibitor (PPI) for acid-related diseases. Despite being a first-line treatment, its overuse due to easy accessibility and lack of public awareness about usage guidelines may lead to potential side effects, necessitating a reassessment of its safety.

**Methods:**

We extracted 119,159 adverse event reports (AERs) related to omeprazole from the FDA Adverse Event Reporting System (FAERS) database, covering data from Q1 2004 to Q4 2023. A disproportionality analysis was performed to evaluate indications, concomitant medication use, and safety.

**Results:**

Omeprazole was commonly prescribed for gastroesophageal reflux disease (GERD), dyspepsia, peptic ulcers, and gastritis. It was frequently used with drugs like aspirin, lisinopril, furosemide, atorvastatin, and metoprolol. Notably, renal and urinary disorders showed strong positive signals, including chronic kidney disease, acute kidney injury, and renal failure, with statistically significant disproportionality measures. The study also identified adverse reactions not listed on drug labels, such as hyperparathyroidism secondary and intentional product misuse.

**Conclusions:**

Our findings provide new insights into the safety of omeprazole in real-world clinical settings, highlighting novel adverse events and offering evidence for safer clinical use.

## Introduction

Peptic ulcer and gastroesophageal reflux disease are both the most common digestive disorders. Peptic ulcers, which occur mainly in the stomach and proximal duodenum, are acidic, digestive injuries to the muscular layer of the mucosa and deeper tissues of the digestive tract caused by a combination of stomach acid and pepsin [1]. The lifetime prevalence of peptic ulcers is around 5–12% [2,3]. In the United States, over 500,000 people are diagnosed with peptic ulcers annually, which places a significant burden on the public health system and government finances [4]. Gastroesophageal reflux disease (GERD) is a condition characterized by the reflux of stomach acid into the esophagus, of which acid reflux and heartburn are the most typical clinical symptoms [5,6]. The prevalence of GERD varies slightly across different countries and regions. In the United States, the prevalence of GERD ranges from approximately 6% to 30%, while in China, it ranges from approximately 2.5% to 29.8% [7]. Proton pump inhibitors (PPIs) and histamine 2 receptor antagonists (H2RAs), two common acid-suppressing drugs, are widely used in the treatment of peptic ulcers and GERD and have been found to be highly effective in clinical practice [8–10].

Omeprazole, a commonly utilized PPI, selectively inhibits the activity of H + /K + ATPase in gastric parietal cells, effectively suppressing gastric acid secretion [11]. Over the past two decades, PPIs have progressively superseded H2RAs as the first-line therapy for acid suppression due to their remarkable acid-inhibitory effects. However, the ease of accessibility of PPIs and the public’s inadequate awareness of their usage restrictions have contributed to the increasingly prominent phenomenon of overuse [12], which has sparked widespread concern and apprehension. In a retrospective study conducted in China, researchers conducted an in-depth analysis of up to 25,850 prescription records, revealing an alarming number of 13,589 prescriptions involving inappropriate prescribing of PPIs, particularly omeprazole [13]. This finding underscores the urgent need for standardizing the use of PPIs and reducing unnecessary prescriptions. Furthermore, numerous studies have corroborated that prolonged and inappropriate use of omeprazole significantly elevates the risk of a myriad of adverse events (AEs) spanning multiple organ systems [14–17]. These risks encompass, but are not limited to, electrolyte imbalances such as hyponatremia [14], skeletal health issues including osteoporosis and heightened fracture risk [15,16], as well as renal impairment and an increased incidence of chronic kidney disease [17]. Consequently, the rational and prudent use of omeprazole is of paramount importance in ensuring patient safety and mitigating medical risks.

The study analyzed AERs related to omeprazole in the FDA Adverse Event Reporting System (FAERS) database using multiple metrics in Disproportionality Analysis (DPA) to explore potential adverse drug reactions (ADRs) signals in a real-world setting.

## Methods

### Sources of omeprazole data

The FAERS (the FDA Adverse Event Reporting System) database was released by the U. S. Food and Drug Administration (FDA) in 2004. Its primary purpose is to collect and analyze AERs for drugs and biologics. The data is updated quarterly to monitor drug safety and identify potential risks to protect public health. This study utilized publicly available anonymized data from the FAERS database. As the data does not contain identifiable information and the research does not involve direct interaction with human participants, ethics approval and informed consent were not required.

Based on the time to market of omeprazole, this retrospective drug safety study downloaded the data on omeprazole-related adverse reactions from the first quarter of 2004 to the fourth quarter of 2023 (a total of 80 quarters) and analyzed the data using R software 4.3.3 and Excel.

### Standardization and extraction of omeprazole data

**Fig 1:**
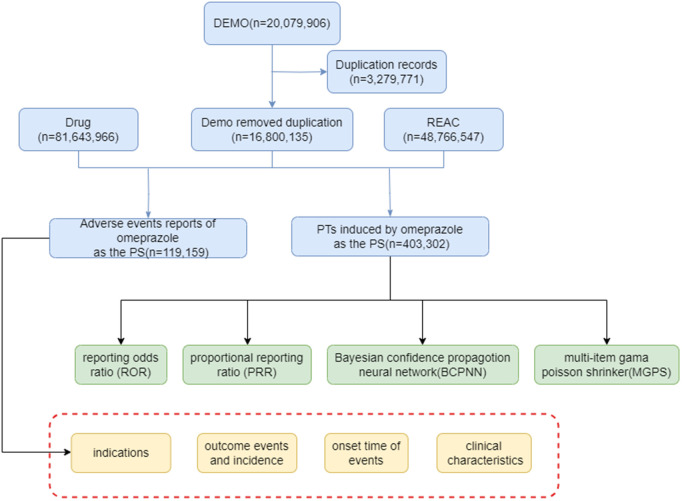
The flow diagram of selecting and analyzing omeprazole-related ADEs from FAERS database.

Reports listing omeprazole as the primary suspect drug were included. To ensure data accuracy and uniqueness, the following FDA-recommended deduplication criteria were applied: (1) Extract the PRIMARYID, CASEID, and FDA_DT fields from the DEMO table of the FAERS database. (2) When CASEID are the same, select the most recent FDA_DT; (3) When both CASEID and FDA_DT are identical, select the PRIMARYID with larger numerical values [18]. Drug names underwent standardization through the application of the Medex_UIMA_1.3.8 system, a natural language processing-based tool specifically engineered to extract and normalize medication information from unstructured textual data, thereby mitigating variability in the reporting of drug nomenclature [19]. MedDRA (Medical Dictionary for Regulatory Activities), serving as a standardized medical terminology system, primarily functions to classify adverse event information. This system maps each individual adverse event to its corresponding Preferred Term (PT) and System Organ Class (SOC). In the present study, we employed MedDRA version 25.0 to conduct the classification of adverse events, ensuring that each adverse event was accurately mapped to its respective PT and the associated SOC [20]. Despite the excellent performance of these tools, Medex UIMA 1.3.8 may misclassify due to text ambiguity, and inconsistencies may arise in MedDRA coding owing to subjectivity. To enhance accuracy, we conducted sampling-based manual reviews. The general flow chart of this study is illustrated in Fig 1.

### Analysis of omeprazole data

DPA is a commonly used method in pharmacovigilance studies to assess the association between drug exposure and adverse effects. In this study, DPA was applied to monitor the drug ADR signals. Common metrics used in DPA include Reported Ratio Ratio (ROR), Proportional Reporting Ratio (PRR), Bayesian Confidence Propagation Neural Network (BCPNN), and Empirical Bayesian Geometric Mean (EBGM) (S1 Fig). These metrics are standard in pharmacovigilance research, particularly when analyzing data from spontaneous reporting systems like the FAERS [21–23]. The ROR is a highly sensitive measure that can identify even weak associations between drugs and adverse reactions. PRR considers the background reporting rate and eliminates any effects that may be caused by factors such as reporting bias. BCPNN can handle parameter uncertainty and provide more reliable predictions. EBGM reduces bias caused by insufficient or unevenly distributed data, resulting in more reliable estimation results. By using a 2 × 2 table to calculate multiple indicators as described above, the results can be cross-checked, enhancing their reliability (S1 Table). We selected AEs signals that simultaneously satisfied the criteria of all four algorithms at the PT level. By employing these four metrics, we ensure a multifaceted assessment of the association between omeprazole and AEs, enhancing their reliability, which is a standard practice to increase the confidence in detected signals [24]. This multi-indicator approach enhances the reliability of the detected signals, which is particularly crucial in pharmacovigilance studies where data quality and variability may be substantial.

## Results

### Basic demographic characteristics

**Fig 2:**
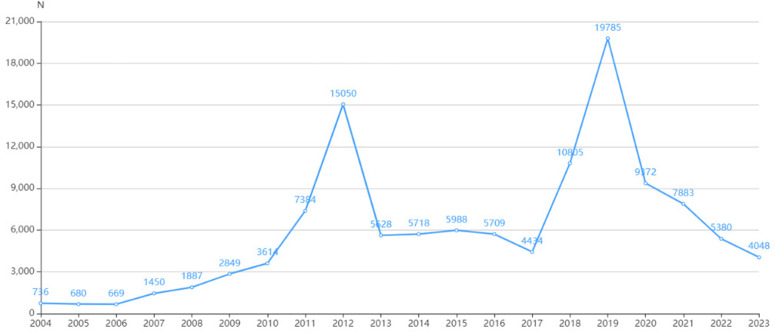
The number of AERs per year since the launch of omeprazole. The X-axis represents the timeline of drug use, and the Y-axis shows the number of reports per quarter.

An analysis of the FEARS database from Q1 2004 to Q4 2023 idenfied 16,800,135 AERs, of which 119,159 were related to omeprazole, involving a total of 403,302 PTs. The highest number of AERs occurred in 2019 with 19,785 reports (16.60% of the total), followed by 2012 with 15,050 reports (12.63%) (Fig 2). A global distribution of omeprazole AERs revealed that the United States had the highest concentration of AERs, accounting for 55.67% of the total number of reports (S2 Table). This figure is noteworthy as it reflects the prominence of the United States in the monitoring of adverse drug reactions to omeprazole.

Of the reported cases, female patients accounted for 54.51%, slightly more than male patients at 31.29%. The median age of the individuals reporting these AEs was 60.32 years. Additionally, the median weight of individuals reporting these AEs was 77.11 kilograms. Excluding unknown data (26.75%), consumers were the most prevalent reporter type, accounting for 41.08%, followed by physician and lawyer with 10.47% and 8.36%, respectively. Oral administration accounted for the highest proportion among those with a clearly specified route, reaching 81.18%. Among the reported outcomes, excluding those with unclear severity (61.92%), hospitalization were the most frequent consequence, accounting for 23.66%, followed by death, which constituted 6.95% of the total (Table 1).

**Table 1:** Basic demographic characteristics.

Characteristics	Number of AER (%)
Sex	
Female	64957 (54.51)
Male	37290 (31.29)
Unkown	16912 (14.19)
Age	
Median (Q1,Q3)	60.32 (50.00,71.00)
Weight	
Median (Q1,Q3)	77.11 (64.40,92.50)
Reporter	
Consumer	48953 (41.08)
Unkown	31879 (26.75)
Physician	12472 (10.47)
Lawyer	9962 (8.36)
Other health-professional	8141 (6.83)
Pharmacist	7742 (6.50)
Registered Nurse	10 (0.01)
Route	
Other	96739 (81.18)
Oral	21642 (18.16)
Intravenous	782 (0.66)
Outcomes	
Other serious	59731 (61.92)
Hospitalization	22822 (23.66)
Death	6705 (6.95)
Disability	3478 (3.61)
Life threatening	2984 (3.09)
Required intervention to Prevent Permanent Impairment/Damage	447 (0.46)
Congenital anomaly	294 (0.30)

Excluding unknown Indication (14.55%), A analysis of AERs revealed that the five most common indications for omeprazole were GERD (49.46%), dyspepsia (8.34%), peptic ulce, gastric disorder, and gastritis (4.76%, 3.2%, and 2.6%, respectively). The five most frequently observed drugs shared by omeprazole are aspirin, lisinopril, furosemide, atorvastatin, and metoprolol. (Table 2)

**Table 2:** Top five indications and top five concomitant medications of Omeprazole.

	Omeprazole	N(%)
**Indications**	Gastrooesophageal Reflux Disease	33920 (49.46)
	Unknown Indication	9980 (14.55)
	Dyspepsia	5718 (8.34)
	Peptic Ulcer	3267 (4.76)
	Gastric Disorder	2198 (3.2)
	Gastritis	1782 (2.6)
**Concomitant medications**	Aspirin	13714
	Lisinopril	9916
	furosemide	9619
	Atorvastatin	9547
	Metoprolol	7508

### Signal analysis of SOCs

Through statistical analysis of AERs related to omeprazole, we identified 24 SOCs associated with omeprazole-related AERs (Table 3). Of these, a total of four SOCs were found to meet at least one of the four algorithmic criteria applied. These SOCs were classified as renal and urinary disorders (case reports = 86330; ROR (95% CI) = 15.02(14.9, 15.14); PRR (95% CI) = 12.02(12.02, 12.02); IC (IC025) = 3.46(3.45); and EBGM (EBGM05) = 11.02(10.94)), gastrointestinal disorders (case reports = 66001; ROR (95% CI) = 2.01(1.99, 2.03); PRR (95% CI) = 1.84(1.84, 1.84); IC (IC025) = 0.87(0.86); and EBGM (EBGM05) = 1.83(1.82)), metabolism and nutrition disorders (case reports = 13289; ROR (95% CI) = 1.49(1.46, 1.51); PRR (95% CI) = 1.47(1.44, 1.5); IC (IC025) = 0.55(0.53); and EBGM (EBGM05) = 1.46(1.44)) and endocrine disorders (case reports = 2944; ROR (95% CI) = 2.84(2.74, 2.95); PRR (95% CI) = 2.83(2.72, 2.94); IC (IC025) = 1.48(1.42); and EBGM (EBGM05) = 2.78(2.7)). Furthermore, only renal and urinary disorders met four criteria and were the most common SOC involved in AEs.

**Table 3:** Signal analysis of SOCs.

SOCs	Case Reports	ROR (95% CI)	PRR (95% CI)	IC (IC025)	EBGM (EBGM05)
renal and urinary disorders	86330	15.02 (14.9, 15.14)	12.02 (12.02, 12.02)	3.46 (3.45)	11.02 (10.94)
gastrointestinal disorders	66001	2.01 (1.99, 2.03)	1.84 (1.84, 1.84)	0.87 (0.86)	1.83 (1.82)
general disorders and administration site conditions	51267	0.65 (0.65, 0.66)	0.7 (0.7, 0.7)	−0.51 (−0.53)	0.7 (0.69)
injury, poisoning and procedural complications	28540	0.73 (0.72, 0.74)	0.75 (0.74, 0.76)	−0.41 (−0.43)	0.75 (0.75)
nervous system disorders	22937	0.61 (0.6, 0.62)	0.63 (0.62, 0.64)	−0.65 (−0.67)	0.64 (0.63)
musculoskeletal and connective tissue disorders	22367	1 (0.99, 1.02)	1 (0.98, 1.02)	0 (−0.02)	1 (0.99)
psychiatric disorders	16724	0.67 (0.66, 0.68)	0.68 (0.67, 0.69)	−0.54 (−0.57)	0.69 (0.68)
investigations	15557	0.57 (0.57, 0.58)	0.59 (0.58, 0.6)	−0.75 (−0.78)	0.59 (0.58)
respiratory, thoracic and mediastinal disorders	15387	0.75 (0.74, 0.77)	0.76 (0.75, 0.78)	−0.39 (−0.41)	0.77 (0.75)
metabolism and nutrition disorders	13289	1.49 (1.46, 1.51)	1.47 (1.44, 1.5)	0.55 (0.53)	1.46 (1.44)
skin and subcutaneous tissue disorders	12143	0.52 (0.51, 0.53)	0.54 (0.53, 0.55)	−0.89 (−0.92)	0.54 (0.53)
infections and infestations	9441	0.41 (0.41, 0.42)	0.43 (0.42, 0.44)	−1.22 (−1.25)	0.43 (0.42)
cardiac disorders	7801	0.68 (0.66, 0.69)	0.68 (0.67, 0.69)	−0.55 (−0.58)	0.68 (0.67)
blood and lymphatic system disorders	6677	0.93 (0.91, 0.96)	0.93 (0.91, 0.95)	−0.1 (−0.13)	0.93 (0.92)
vascular disorders	5338	0.57 (0.56, 0.59)	0.58 (0.57, 0.59)	−0.79 (−0.82)	0.58 (0.57)
neoplasms benign, malignant and unspecified (incl cysts and polyps)	5036	0.43 (0.42, 0.44)	0.44 (0.43, 0.45)	−1.18 (−1.22)	0.44 (0.43)
eye disorders	4621	0.54 (0.53, 0.56)	0.55 (0.54, 0.56)	−0.86 (−0.9)	0.55 (0.54)
immune system disorders	3489	0.75 (0.72, 0.77)	0.75 (0.72, 0.78)	−0.41 (−0.46)	0.75 (0.73)
hepatobiliary disorders	3144	0.82 (0.79, 0.84)	0.82 (0.79, 0.85)	−0.29 (−0.34)	0.82 (0.79)
endocrine disorders	2944	2.84 (2.74, 2.95)	2.83 (2.72, 2.94)	1.48 (1.42)	2.78 (2.7)
ear and labyrinth disorders	1579	0.87 (0.82, 0.91)	0.87 (0.82, 0.92)	−0.21 (−0.28)	0.87 (0.83)
reproductive system and breast disorders	1441	0.4 (0.38, 0.43)	0.41 (0.39, 0.43)	−1.29 (−1.37)	0.41 (0.39)
congenital, familial and genetic disorders	668	0.5 (0.47, 0.54)	0.51 (0.47, 0.55)	−0.98 (−1.09)	0.51 (0.48)
pregnancy, puerperium and perinatal conditions	581	0.31 (0.29, 0.34)	0.31 (0.29, 0.34)	−1.67 (−1.78)	0.31 (0.29)

### Signal analysis of PTs

This study analyzed adverse drug reactions using four indicators and screened a total of 428 PTs that met the four assessment criteria (S3 Table). We selected the top 20 adverse reactions by ranking according to the number of case reports and categorized by the system (Table 4).

**Table 4:** PTs by ranking according to the number of case reports.

SOCs	PTs	Case Reports	ROR(95% CI)	PRR(95% CI)	IC(IC025)	EBGM(EBGM05)
renal and urinary disorders	chronic kidney disease	28639	87.19 (85.87, 88.54)	81.07 (79.5, 82.67)	5.61 (5.59)	48.77 (48.15)
	acute kidney injury	15935	18.54 (18.22, 18.85)	17.84 (17.49, 18.19)	3.97 (3.94)	15.66 (15.44)
	renal failure	13224	15.16 (14.89, 15.44)	14.7 (14.41, 14.99)	3.72 (3.7)	13.2 (13)
	end stage renal disease	8516	123.18 (119.51, 126.96)	120.6 (115.96, 125.42)	5.92 (5.88)	60.63 (59.12)
	renal injury	8208	49.73 (48.46, 51.03)	48.74 (47.79, 49.7)	5.13 (5.09)	34.94 (34.2)
	tubulointerstitial nephritis	3985	41.63 (40.16, 43.16)	41.23 (39.65, 42.88)	4.95 (4.9)	30.94 (30.02)
gastrointestinal disorders	gastrooesophageal reflux disease	9705	20.76 (20.32, 21.22)	20.29 (19.9, 20.69)	4.13 (4.1)	17.5 (17.18)
	dyspepsia	5882	9.44 (9.19, 9.7)	9.32 (9.14, 9.5)	3.12 (3.09)	8.72 (8.53)
	rebound acid hypersecretion	2534	638.39 (578.97, 703.92)	634.39 (575.17, 699.71)	6.67 (6.59)	101.69 (93.71)
	hyperchlorhydria	2515	98.78 (93.7, 104.14)	98.18 (92.57, 104.13)	5.77 (5.7)	54.43 (52.08)
	flatulence	1257	3.29 (3.11, 3.48)	3.28 (3.09, 3.48)	1.69 (1.61)	3.22 (3.07)
blood and lymphatic system disorders	nephrogenic anaemia	2914	229.15 (215.4, 243.78)	227.5 (214.51, 241.28)	6.31 (6.24)	79.18 (75.19)
injury, poisoning and procedural complications	intentional product misuse	2795	4.8 (4.62, 4.98)	4.77 (4.59, 4.96)	2.21 (2.16)	4.63 (4.48)
	multiple fractures	1486	13.02 (12.34, 13.74)	12.98 (12.24, 13.77)	3.56 (3.48)	11.81 (11.29)
musculoskeletal and connective tissue disorders	osteoporosis	2654	9.66 (9.29, 10.06)	9.61 (9.24, 9.99)	3.17 (3.11)	8.97 (8.68)
general disorders and administration site conditions	rebound effect	2138	51.43 (48.89, 54.11)	51.16 (48.24, 54.26)	5.18 (5.11)	36.16 (34.66)
metabolism and nutrition disorders	hypomagnesaemia	1911	24.53 (23.35, 25.77)	24.42 (23.03, 25.9)	4.35 (4.28)	20.46 (19.63)
	hyponatraemia	1305	3.32 (3.14, 3.51)	3.31 (3.12, 3.51)	1.7 (1.62)	3.25 (3.1)
	hypocalcaemia	1220	10.04 (9.47, 10.65)	10.01 (9.44, 10.62)	3.22 (3.14)	9.32 (8.87)
endocrine disorders	hyperparathyroidism secondary	1489	203.89 (187.57, 221.63)	203.14 (187.82, 219.71)	6.25 (6.15)	76.03 (70.91)

### Analysis of safety signals in system organ class

**Fig 3:**
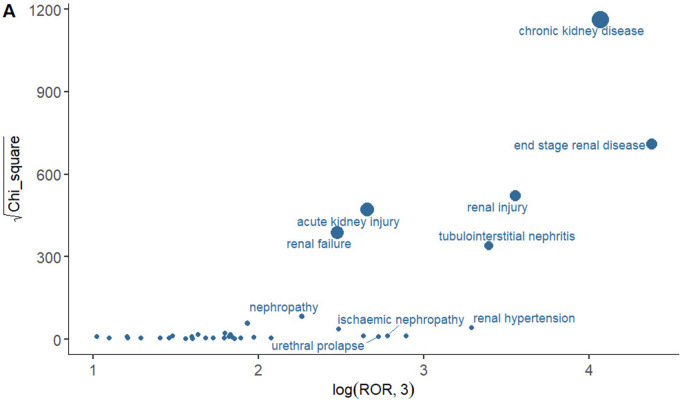
Analysis of safety signals in system organ class. The x-axis displays the log3ROR value, the y-axis represents the square root of the χ^2^ value, and the size of the points in the graph reflects the number of adverse reactions reported.

Among all the SOCs, renal and urologic disorders were the only ones to satisfy the four calculation criteria and were also the most prevalent SOC. Consequently, we undertook signal strength calculations for the associated AEs occurring within this SOC. Fig 3 visualizes the signal intensity of AEs using the logarithmically transformed reporting odds ratio (log3ROR, X-axis) and the square root of the chi-square statistic (square root χ², Y-axis). By applying a logarithmic transformation to the ROR, log3ROR renders the data distribution more stable. Higher log3ROR values indicate a stronger association between the drug and the AE. The square root χ² measures the statistical significance of the signal, and the square root transformation compresses the range of the chi-square statistic, making the chart easier to interpret. The size of the dots in the figure represents the number of adverse event reports, and the farther a dot is from the origin, the higher the signal intensity. In this SOC, the top three signal strengths were chronic kidney disease, end stage renal disease and renal injury. We provide explanations of each element in Fig 3 in S4 Table to facilitate the visualization of Fig 3.

## Discussion

Omeprazole, a commonly prescribed PPI, functions by inhibiting the “proton pump” (H + /K + ATPase) of parietal cells in the stomach, thereby diminishing gastric acid secretion [25]. Its widespread use stems from its remarkable acid-suppressing capability. Given its efficacy and safety profile in the majority of cases, off-label utilization of omeprazole is prevalent [26]. However, with the increasing usage of medications and the diversity of clinical indications among patients, drug-related adverse reactions and their indications are subject to change, underscoring the need for renewed evaluations. This study analyzed omeprazole-associated AERs and indications based on real-world data in order to assess its safety and efficacy and provide a safety reference for clinical use.

A comprehensive analysis of the FAERS database between the first quarter of 2004 and the fourth quarter of 2023 revealed a total of 119,159 AERs involving omeprazole. Of these, 54.51% were reported by women, whcich was higher than the 31.29% reported by men. While multiple studies have indicated a higher incidence of GERD in males compared to females [27,28], it is noteworthy that specific research has revealed that females are approximately 40% more likely to exhibit symptoms of GERD than males [7]. This phenomenon may account for the tendency of females to use omeprazole more frequently due to the more pervasive impacts of GERD symptoms they experience, subsequently contributing to a higher proportion of AERs associated with omeprazole among females. In terms of reporter types, AERs of omeprazole are primarily sourced from consumers. This phenomenon can be attributed to the drug’s high degree of popularization and ease of access, as well as a notable increase in consumers’ awareness regarding self-monitoring of their health status. Furthermore, the United States is the primary source country for reports, which may be related to the country’s well-established drug regulation and adverse reaction reporting system. This system facilitates the reporting and tracking of adverse reactions to drugs by healthcare organizations, pharmaceutical companies, researchers, and the public.

In our study, we identified the five most frequently co-prescribed medications with omeprazole as aspirin, lisinopril, furosemide, atorvastatin, and metoprolol. Existing literature suggests that omeprazole may enhance the cholesterol-lowering effects of atorvastatin through competitive inhibition of CYP3A4 and CYP2C19 metabolism [29]. However, this interaction is associated with potential risks, including an increased incidence of adverse events such as myalgia and liver injury in patients [30]. Aspirin is commonly administered concomitantly with omeprazole to mitigate the risk of gastrointestinal bleeding [31]. Regarding lisinopril, furosemide, and metoprolol, the current body of literature does not highlight direct interactions with omeprazole. Consequently, future research endeavors could delve deeper into the interactions between omeprazole and these commonly co-prescribed medications, particularly their influence on adverse event profiles.

Our research has revealed that omeprazole has been associated with AEs across a total of 24 SOCs. It is noteworthy that three of these SOCs—the neoplasms benign, malignant and unspecified (incl cysts and polyps), congenital, familial and genetic disorders, and pregnancy, puerperium and perinatal conditions—are not currently mentioned in the existing drug labels, which suggests the potential for unrecognized risks.Among the 24 SOCs, the renal and urinary disorders consistently demonstrated strong positive signals across four distinct signals (case reports = 86330; ROR (95% CI) = 15.02(14.9, 15.14); PRR (95% CI) = 12.02(12.02, 12.02); IC (IC025) = 3.46(3.45); and EBGM (EBGM05) = 11.02(10.94)). Furthermore, the endocrine disorders exhibited strong positive signals in three signals (case reports = 2944; PRR (95% CI) = 2.83(2.72, 2.94); IC (IC025) = 1.48(1.42); and EBGM (EBGM05) = 2.78(2.7)), while the gastrointestinal disorders (case reports = 66001; IC (IC025) = 0.87(0.86)) and metabolism and nutrition disorders (case reports = 13289; IC (IC025) = 0.55(0.53)) also showed strong positive signals in individual signals. In contrast, the signals for the remaining SOCs were not statistically significant.

Our study was centered on the top 20 most frequent PTs, which were extensively distributed across eight distinct SOCs. Notably, PTs associated with the renal and urinary disorders constituted a significant proportion, accounting for up to 30%, closely followed by those related to the gastrointestinal disorders, comprising 25% of the total.

Among the PTs within the renal and urinary disorders, tubulointerstitial nephritis has been explicitly listed in drug product labels, and multiple studies have established a close association between the use of omeprazole and the occurrence of tubulointerstitial nephritis [32–34]. Although the precise mechanisms underlying this association remain elusive, research has hinted at the activation of interleukin-17, T helper (Th)-1, and Th-17-mediated immune-inflammatory responses, primarily localized to renal tubules with minimal impact on glomeruli [35]. Nevertheless, it is noteworthy that other severe renal complications potentially induced by omeprazole, such as chronic kidney disease, acute kidney injury, renal failure, end stage renal disease, and renal impairment, are conspicuously absent from drug product labels. Several studies have unveiled that these adverse effects may stem from omeprazole-induced oxidative stress and cell death in renal tubular cells [36]. Furthermore, the resultant renal insufficiency can exacerbate the development of renal anemia [37], a pertinent concern in the blood and lymphatic system disorders. While anemia as a general effect is acknowledged in drug product labels, the renal anemia by renal insufficiency is not mentioned.

Within the gastrointestinal disorders, dyspepsia and flatulence, as common PTs, have been explicitly noted in the drug product labels. Prior clinical trials have robustly demonstrated that PPIs, such as omeprazole, can elicit clinical manifestations including dyspepsia and bloating [38]. This phenomenon may be underpinned by a marked increase in small intestinal bacterial overgrowth during PPI usage [39,40], which serves as a pivotal factor contributing to the development of dyspepsia and flatulence [41]. Furthermore, although conditions like GERD, rebound acid hypersecretion, and hyperchlorhydria may not be directly mentioned in the drug product labels, omeprazole, being the first-line treatment for GERD, can potentially induce hypergastrinemia during its administration. This, in turn, may precipitate rebound acid hypersecretion and hyperchlorhydria, ultimately exacerbating or triggering GERD [17]. Animal studies have shed light on the possible mechanisms underlying this scenario: omeprazole administration may be accompanied by hyperplasia of enterochromaffin-like cells, which is intimately associated with severe hypergastrinemia [42]. At last severe hypergastrinemia facilitates the occurrence of rebound acid hypersecretion and hyperchlorhydria [43].

In the metabolism and nutrition disorders, the drug product labels of omeprazole has noted the potential association of hypomagnesaemia, hyponatraemia, and hypocalcaemia with its use, and numerous studies have corroborated the induction of these three PTs by omeprazole [44–46]. The occurrence of hypomagnesaemia may be attributed to the inhibition of gastric acid secretion by proton PPIs, leading to an elevation in luminal pH, which subsequently decreases the activity of TRPM6 and reduces magnesium absorption [47]. As for hyponatraemia, its mechanism may be linked to fluid retention resulting from the syndrome of inappropriate antidiuretic hormone secretion induced by omeprazole [48]. In the case of hypocalcaemia, omeprazole may interfere with the acidified environment of bone resorption lacunae, causing inactivation of lysosomal enzymes such as tartrate-resistant acid phosphatase (TRAP), thereby slowing down bone matrix degradation and bone resorption processes [49]. Notably, although osteoporosis and hyperparathyroidism secondary are not mentioned in the drug product labels of omeprazole, multiple fractures and hypocalcaemia are explicitly noted. However, studies have demonstrated that all four conditions are PTs associated with omeprazole and are interlinked [46,50–52]. Specifically, multiple fractures, osteoporosis, and hyperparathyroidism secondary are closely related to hypocalcaemia. The administration of omeprazole disrupts the acidification of lacunae during the bone resorption process, inhibiting the activity of lysosomal enzymes such as TRAP. This inhibition leads to a reduction in bone matrix degradation and bone resorption, subsequently inducing hypocalcaemia. In turn, hypocalcaemia further promotes the development of hyperparathyroidism secondary, exacerbates osteoporosis, and elevates the risk of fractures [49].

In the general disorders and administration site conditions, while rebound effects are not explicitly detailed in the medication’s package insert, they can be regarded as equivalent to the phenomenon of rebound acid hypersecretion. Furthermore, the accessibility of omeprazole, combined with its widespread perception of safety, and lack of significant side effects, contributes to the potential for intentional product misuse, an aspect that is similarly omitted from the drug product labels.

Our study is subject to several limitations. Firstly, a constraint inherent in the FAERS lies in the predominantly self-reported nature of its data, rendering the data susceptible to various external influences and inevitably introducing biases that are difficult to fully eliminate. Furthermore, the significant disparity in the number of reports submitted by consumers versus physician may lead to inaccuracies in the depiction of PTs. Additionally, the lack of detailed information on the severity of PTs in the FAERS database poses a limitation on our capacity to conduct more profound research into PTs. It is also noteworthy that the use of DPA prevents us from establishing a direct causal relationship between drugs and PTs, instead merely revealing statistical correlations between them. Consequently, to more precisely elucidate the relationship between drugs and PTs, conducting more rigorous and prospective studies remains crucial.

## Conclusion

This study conducted a comprehensive and meticulous analysis of the indications, concomitant medication use, and AEs associated with omeprazole utilizing the FAERS. The common indications for omeprazole encompass GERD, dyspepsia, peptic ulce, gastric disorder and gastritis. In terms of concomitant medication, omeprazole is frequently administered in combination with aspirin, lisinopril, furosemide, and other drugs. The results of this study revealed that many PTs align with the descriptions provided in the drug product labels. However, it is noteworthy that omeprazole-induced PTs related to the renal and urinary disorders warrant significant attention. These renal-related PTs, such as chronic kidney disease, acute kidney injury, renal failure and end stage renal disease, are largely unmentioned in the drug product labels. This finding underscores the need for healthcare professionals to remain vigilant regarding such risks. Furthermore, the prominent intentional product misuse of omeprazole necessitates the urgent implementation of further regulatory measures.

## Supporting information

S1 Fig Commonmetrics. (TIF)

S1 Table Four grid table. (DOCX)

S2 Table A regional analysis of AERs for omeprazole. (DOCX)

S3 Table 428 PTs that met the four assessment criteria. (XLSX)

S4 Table Explanations of each element in Fig 3. (DOCX)

## Abbreviations

FAERS: FDA Adverse Event Reporting System
ROR: ratio of ratios, PRR: proportional reporting ratio
BCPNN: Bayesian Confidence Propagation Neural Network
MGPS: Multi-Item Gamma Poisson Shrinker
SOC: System Organ Class
PT: preferred terms
ADE: adverse drug event
AERs: adverse event reports
PPI: proton pump inhibitor
GERD: gastroesophageal reflux disease
H2RAs: histamine 2 receptor antagonists.
