# Supplementary figures and images for: Updating understanding of real-world adverse events associated with omeprazole
Source: PLoS One. 2025 Aug 20;20(8):e0330509. doi: 10.1371/journal.pone.0330509 (PMC12367145; doi:10.1371/journal.pone.0330509)

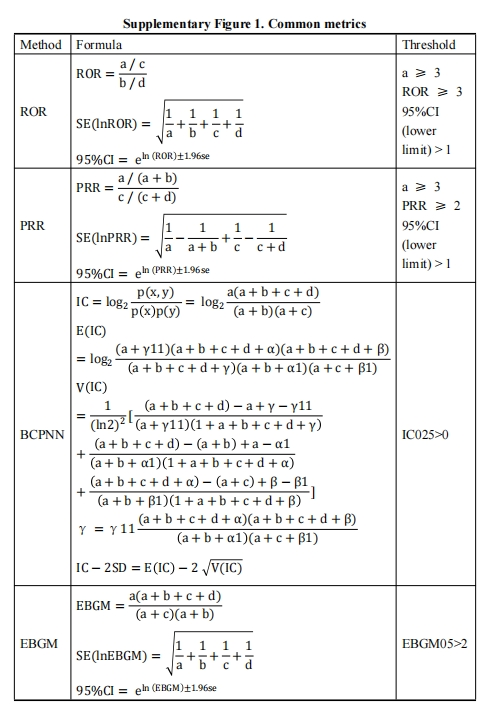

Supplement: S1 Fig — (TIF) [file pone.0330509.s001.tif]
