# Supplementary material for: Updating understanding of real-world adverse events associated with omeprazole
Source: PLoS One. 2025 Aug 20;20(8):e0330509. doi: 10.1371/journal.pone.0330509 (PMC12367145; doi:10.1371/journal.pone.0330509)
Supplement: S1 Table — (DOCX) [file pone.0330509.s002.docx]

| **Supplementary Table 1. Four grid table** | | | |
| --- | --- | --- | --- |
|  | Drug-related AEs | Non-drug-related AEs | Total |
| Drug | a | b | a + b |
| Non-drug | c | d | c + d |
| Total | a + c | b + d | N = a + b + c + d |
