# Supplementary material for: Updating understanding of real-world adverse events associated with omeprazole
Source: PLoS One. 2025 Aug 20;20(8):e0330509. doi: 10.1371/journal.pone.0330509 (PMC12367145; doi:10.1371/journal.pone.0330509)
Supplement: S2 Table — (DOCX) [file pone.0330509.s003.docx]

| **Supplementary Table 2. A regional analysis of AERs for omeprazole.** | |
| --- | --- |
| **Characteristics** | **Number of AER (%)** |
| Reported countries |  |
| United States | 66341 (55.67) |
| other | 31340(26.30) |
| United Kingdom | 6664( 5.59) |
| France | 4584( 3.85) |
| Spain | 1616( 1.36) |
| Japan | 1086( 0.91) |
| Canada | 1028( 0.86) |
| Netherlands | 904( 0.76) |
| Germany | 814( 0.68) |
| Italy | 776( 0.65) |
| Sweden | 618( 0.52) |
| Brazil | 544( 0.46) |
| China | 522( 0.44) |
| Australia | 308( 0.26) |
| Portugal | 273( 0.23) |
| Switzerland | 272( 0.23) |
| South Africa | 234( 0.20) |
| Poland | 201( 0.17) |
| Belgium | 137( 0.11) |
| India | 103( 0.09) |
| Russia | 101( 0.08) |
| Denmark | 97( 0.08) |
| Ireland | 93( 0.08) |
| Singapore | 85( 0.07) |
| Israel | 73( 0.06) |
| Greece | 63( 0.05) |
| Romania | 63( 0.05) |
| Chile | 62( 0.05) |
| Norway | 57( 0.05) |
| Czechia | 50( 0.04) |
