# Supplementary material for: Updating understanding of real-world adverse events associated with omeprazole
Source: PLoS One. 2025 Aug 20;20(8):e0330509. doi: 10.1371/journal.pone.0330509 (PMC12367145; doi:10.1371/journal.pone.0330509)
Supplement: S4 Table — (DOCX) [file pone.0330509.s005.docx]

| **Supplementary Table 4. Explanations of each element in Figure 3** | |
| --- | --- |
| Elements | Explanations |
| X-axis (log3ROR) | Measuring the strength of association between drugs and adverse events |
| Y-axis (square root χ²) | Evaluating the statistical significance of the signal |
| Data point | The size of the dots reflects the number of reports. |
